# Draft Genome Sequence of Phenol-Degrading Variovorax boronicumulans Strain HAB-30

**DOI:** 10.1128/MRA.01478-19

**Published:** 2020-02-13

**Authors:** Fatma Azwani Abdul Aziz, Kenshi Suzuki, Ryota Moriuchi, Hideo Dohra, Yosuke Tashiro, Hiroyuki Futamata

**Affiliations:** a Institute of Tropical Agriculture and Food Security, Universiti Putra Malaysia, Serdang, Selangor, Malaysia; b Research Institute of Green Science and Technology, Shizuoka University, Hamamatsu, Shizuoka, Japan; c Department of Applied Chemistry and Biochemical Engineering, Faculty of Engineering, Shizuoka University, Hamamatsu, Shizuoka, Japan; d Graduate School of Science and Technology, Shizuoka University, Hamamatsu, Shizuoka, Japan; Queens College

## Abstract

We report the draft genome sequence of Variovorax boronicumulans strain HAB-30, which was isolated from a phenol-degrading chemostat culture. This strain contains genes encoding a multicomponent type of phenol hydroxylase, with degradation pathways for catechol and other aromatic compounds. The genome sequence will be useful for understanding the metabolic pathways involved in phenol degradation.

## ANNOUNCEMENT

The *Variovorax* genus comprises diverse species that are often found in contaminated environments (1, 2). *Variovorax* strains utilize not only simple carbon sources such as succinate and gluconate but also recalcitrant chemicals such as aromatic sulfonates and polychlorinated biphenyls (3). A chemostat culture was constructed with phenol as the sole carbon and energy source and trichloroethene (TCE)-contaminated aquifer soil as an inoculum (4). After the culture became stable (with respect to the phenol concentration, optical density at 660 nm, and dissolved oxygen concentration), a small portion of the culture was sampled, diluted, and streaked onto agar plates containing MP medium (5) supplemented with 0.5 mM phenol (4). One of the colonies that appeared on the plate was strain HAB-30. Strain HAB-30 was incubated aerobically at 25°C in MP medium supplemented with 0.5 mM phenol. Strain HAB-30 was identified as a member of the *Variovorax* genus by a homology search of 16S rRNA nucleotide sequences using the Basic Local Alignment Search Tool (4). It was reported previously that strain HAB-30 exhibits the greatest TCE-degrading activity, via cometabolism of phenol, among phenol-degrading bacteria (6). Hence, in order to understand the genetic basis of phenol degradation and other degrading abilities of this strain, genome sequence analysis of strain HAB-30 was conducted.

The genomic DNA of Variovorax boronicumulans strain HAB-30 was extracted by a method reported previously (4) and was fragmented using a Covaris acoustic solubilizer. A paired-end library was constructed with the TruSeq DNA PCR-free library preparation kit and was sequenced using the Illumina MiSeq platform (300-bp paired-end reads). Adapter sequences and low-quality ends (with a quality score of <15) of the raw reads were trimmed using Trimmomatic version 0.36 (7). The resultant 2,720,645 paired-end reads (a total of 1,418.6 Mb, with 211.7-fold coverage of the genome) were assembled with SPAdes version 3.13.0 (8), with the options of careful, only assembler, and coverage cutoff of 50 and with default settings. Thirteen contigs were obtained, with an *N*_50_ value of 4,303,337 bp and an average G+C content of 68.4%. The draft genome sequence of strain HAB-30 had a total length of 6,699,939 bp.

Annotation/reannotation of genome sequences was performed for prediction of open reading frames, rRNA genes, and tRNA genes with the DDBJ Fast Annotation and Submission Tool (DFAST-core standalone program version 1.2.0) (9). In total, 6,149 coding regions and 60 tRNAs were predicted and annotated. Average nucleotide identity (ANI) values for the draft genome sequence of V. boronicumulans HAB-30 versus other *Variovorax* spp. were analyzed using JSpeciesWS (10). The highest ANI value was 96.8% with V. boronicumulans strain NBRC 103145^T^ (GenBank accession no. NZ_BCUS00000000) (11).

Strain HAB-30 contained one multicomponent phenol hydroxylase, with *meta*- and *ortho*-cleavage metabolic pathways for catechol via catechol 2,3-dioxygenase and catechol 1,2-dioxygenase, respectively. The biodegradation of aromatic compounds leads to the formation of catechol or its derivatives, which are then transformed into tricarboxylic acid cycle intermediates via *ortho*- or *meta*-cleavage pathways (12, 13). According to the results of annotation, several oxygenases (e.g., protocatechuate 3,4-dioxygenase, *p*-hydroxybenzoate 3-monooxygenase, 4-methoxybenzoate monooxygenase, and functionally unknown monooxygenase and dioxygenase) are found in the genome of strain HAB-30. These findings suggest that strain HAB-30 is a potentially useful bacterium for the bioremediation of aromatic compounds. The genome data will provide a better understanding of phenol-degrading bacteria (14–16), which may contribute to the future design of rational strategies for bioremediation of xenobiotic compounds in the environment.

### Data availability.

The genome sequence of V. boronicumulans HAB-30 has been deposited in DDBJ/ENA/GenBank under accession no. BKDH00000000. The version described in this paper is the first version, BKDH01000000. The raw reads have been deposited in the DDBJ Sequence Read Archive under accession no. DRA008842.
